# A high resolution map of a cyanobacterial transcriptome

**DOI:** 10.1186/gb-2011-12-5-r47

**Published:** 2011-05-25

**Authors:** Vikram Vijayan, Isha H Jain, Erin K O'Shea

**Affiliations:** 1 Graduate Program in Systems Biology, Harvard University, 52 Oxford Street, Northwest 445.40, Cambridge, MA 02138, USA; 2 Howard Hughes Medical Institute, Harvard Faculty of Arts and Sciences Center for Systems Biology, Departments of Molecular and Cellular Biology and Chemistry and Chemical Biology, Harvard University, 52 Oxford Street, Northwest 445.40, Cambridge, MA 02138, USA

## Abstract

**Background:**

Previous molecular and mechanistic studies have identified several principles of prokaryotic transcription, but less is known about the global transcriptional architecture of bacterial genomes. Here we perform a comprehensive study of a cyanobacterial transcriptome, that of *Synechococcus elongatus *PCC 7942, generated by combining three high-resolution data sets: RNA sequencing, tiling expression microarrays, and RNA polymerase chromatin immunoprecipitation sequencing.

**Results:**

We report absolute transcript levels, operon identification, and high-resolution mapping of 5' and 3' ends of transcripts. We identify several interesting features at promoters, within transcripts and in terminators relating to transcription initiation, elongation, and termination. Furthermore, we identify many putative non-coding transcripts.

**Conclusions:**

We provide a global analysis of a cyanobacterial transcriptome. Our results uncover insights that reinforce and extend the current views of bacterial transcription.

## Background

Over the past few decades considerable progress has been made in understanding the mechanisms and regulation of bacterial transcription. However, relatively few studies have attempted to identify the prevalent features of bacterial transcription *de novo *using an unbiased genome-wide approach. This approach to analyzing the bacterial transcriptome may not only help reinforce the progress made from traditional molecular and mechanistic studies, but may also identify new global features in transcription that have previously been underappreciated.

The advent of next-generation sequencing allows for a complete characterization of bacterial genomes that was previously not possible. RNA sequencing gives unprecedented insights into transcription unit architecture, while RNA polymerase chromatin immunoprecipitation (ChIP) sequencing reveals the flow of information into the transcriptome. We provide a comprehensive analysis of a cyanobacterial transcriptome - that of *Synechococcus elongatus *PCC 7942 - integrating data from RNA sequencing, tiling expression microarrays, and RNA polymerase (RNA pol) ChIP sequencing.

The unicellular cyanobacterium *S. elongatus *PCC 7942 is a genetically tractable model organism for prokaryotic photosynthesis [1], bioenergy production, and circadian rhythms [2]. The circadian clock of *S. elongatus *is built on a three-protein central oscillator that controls the global rhythmic expression of the majority of the genome [3,4]. Our transcriptome characterization will facilitate the further use of *S. elongatus *as a model organism.

## Results and discussion

### The transcriptome

We used RNA sequencing, tiling expression microarrays, and RNA pol ChIP sequencing to interrogate transcription in the cyanobacterium *S. elongatus*. RNA was isolated at 4-hour intervals from circadian free-running cells grown in constant light conditions and RNA from a pool of circadian timepoints was sequenced (Materials and methods). Strand-specific RNA sequencing was performed on the Illumina platform yielding over 22 million uniquely mappable non-rRNA reads and over 620 million nucleotides of coverage, strand-specifically covering each nucleotide of the approximately 2.7 Mb genome an average of approximately 115 times [5] (Materials and methods). Agilent two-color microarrays with a total of approximately 488,000 strand-specific 60-nucleotide probes spaced every 12 nucleotides were hybridized with cDNA from individual circadian timepoints to supplement RNA sequencing analysis (Materials and methods). RNA pol ChIP sequencing of subjective dawn and subjective dusk circadian timepoints was performed on the Illumina platform, yielding a total of over 19 million uniquely mappable reads, covering each nucleotide over approximately 1,055 times after extension of reads by 150 bp to cover the average length of sequenced DNA fragments (Materials and methods). All analysis of RNA pol ChIP was performed on the combination of the two circadian timepoints unless otherwise specified.

**Figure 1:**
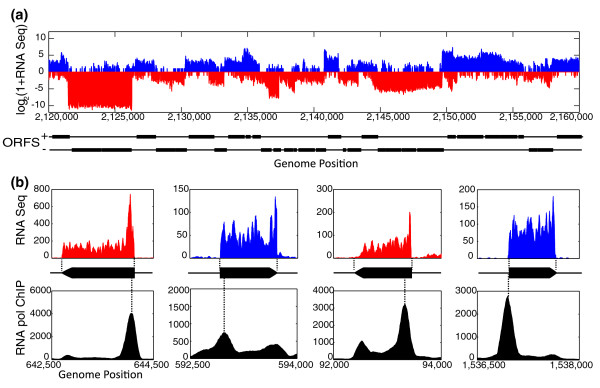
**RNA sequencing and RNA pol ChIP in *S. elongatus***. **(a) **Strand-specific RNA sequencing over a representative 40-kb region in the *S. elongatus *chromosome. Positive strand transcription is shown in blue (positive y-axis), and negative strand transcription in red (negative y-axis). For visualization over full dynamic range, the y-axis shows log_2 _transformed reads per nucleotide of RNA sequencing coverage. The position of Joint Genome Institute predicted ORFs for each strand are shown below in black. High RNA sequencing signal is present at nearly all ORFs and anti-sense transcription is extensive. **(b) **RNA sequencing and RNA pol ChIP sequencing for representative highly expressed transcripts. Top panel: zoomed in view of RNA sequencing coverage of particular mRNA transcripts. Transcripts are color coded by strand as in (a). Transcription units with precise 5' and 3' ends are defined from RNA sequencing data for all mRNAs (black arrow) (Figure S1 in Additional file 2; Materials and methods). Bottom panel: RNA pol ChIP sequencing associated with the transcripts from the top panel. The y-axis is normalized such that the genome average is 200 units per nucleotide. Peaks in RNA pol occupancy are often found near the 5' end of the transcript and occasionally smaller peaks in RNA pol occupancy are located near the 3' ends or inside the transcript. 5' peaks tend to be located within the transcript as opposed to within the promoters.

The RNA sequencing and RNA pol ChIP sequencing profiles demonstrate that the transcription landscape in *S. elongatus *is rather dense with very small inter-transcript regions (Figure 1a). Assuming a relatively strict cutoff of at least two reads per nucleotide for transcription, approximately 88% of the genome is transcribed on either the plus or minus strand, and approximately 55% of each strand is transcribed (Materials and methods). Approximately 82% of all non-coding sequence is transcribed on either the plus or minus strand, highlighting the density of transcription in *S. elongatus*. Fewer than 10% of the 2,612 chromosomally encoded Joint Genome Institute (JGI) predicted ORFs have negligible transcription (less than a mean of two reads per nucleotide across the ORF), and the remaining ORFs have absolute expression distributed over a dynamic range of nearly 10,000. In this study we only sample standard exponential growth conditions during circadian free-run in constant light conditions; both transcription density and the number of expressed ORFs are likely to be higher if multiple growth conditions are sampled.

RNA sequencing affords high-resolution determination of the 5' and 3' ends of each transcription unit. Transcription units were defined using *a priori *knowledge of JGI ORF, tRNA, and rRNA annotations (Materials and methods). A total of 1,473 transcription units were identified, 1,415 of which were designated as mRNA transcripts as they are devoid of tRNA or rRNA and contain at least one JGI annotated ORF. 5' and 3' ends were determined for all transcripts and all subsequent analysis is performed on the subset defined as mRNA transcripts (Table S1 in Additional file 1, Figure S1 in Additional file 2; Materials and methods). Highly expressed transcripts show particularly clear 5' and 3' boundaries of transcription, each with an associated peak in RNA pol occupancy as measured by RNA pol ChIP (Figure 1b). The RNA pol ChIP data are characterized by the presence of several large peaks that tend to be located near the 5' end of transcripts, and many smaller peaks that tend to be located either at the 3' end of highly expressed transcripts or within transcripts (Figure S2 in Additional file 2). Surprisingly, most 5' RNA pol peaks are situated within the transcript rather than at the promoter. Sequence analysis of RNA pol peak positions reveals enrichment for the central AT nucleotides of the highly iterated palindrome 1 (HIP1) site, 5' GCGATCGC 3', at the RNA pol peak maximum (*P *< 1e-10, binomial cumulative distribution). The HIP1 palindrome is highly over-represented in many cyanobacteria, including *S. elongatus - *it appears 185 times more frequently in the *S. elongatus *chromosome than expected for a random 8-mer sequence, but its function is unknown [6]. It is known that the HIP1 motif is a target of methylation in some cyanobacteria [7], raising the possibility of an intriguing link between DNA methylation and transcription. Although RNA pol peaks are enriched at the HIP1 site, fewer than 1% of HIP1 sites (41 of 7,402) are situated at an RNA pol peak, and fewer than 2% of RNA pol peaks (41 of 2,159) are situated at HIP1 sites. Despite the fact that only 41 HIP1 sites are occupied by RNA pol, the probability of having at least this many sites occupied by chance is less than 1e-10 (binomial cumulative distribution).

**Figure 2:**
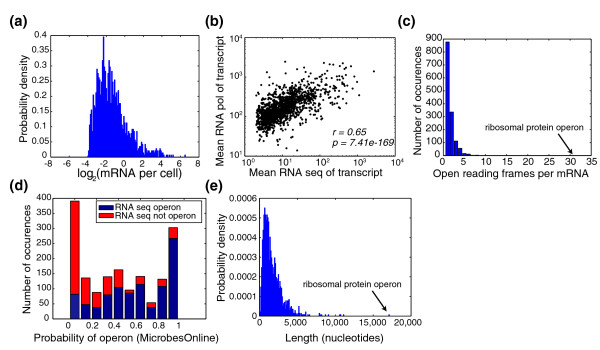
**Basic features of the *S. elongatus *transcriptome**. **(a) **Distribution of absolute transcript abundance per cell. Only transcripts with mean coverage of over two reads per nucleotide (corresponding to approximately 1 mRNA per 15 cells) are shown, and a total of 1,500 mRNA per cell is assumed [8,9] (Materials and methods). **(b) **RNA sequencing versus RNA pol ChIP. Absolute transcription (RNA sequencing averaged over transcript) and absolute RNA pol occupancy (RNA pol ChIP averaged over transcript) are generally correlated (Pearson correlation, r = 0.65). The probability of getting a correlation as large by random chance (*P*-value) is 7.41e-169. **(c) **Distribution of ORFs per mRNA. Most mRNAs contain one to two ORFs. The extreme case is that of an operon composed primarily of ribosomal proteins that includes 31 ORFs and is 17,158 nucleotides in length. **(d) **Operon estimations based on RNA sequencing versus bioinformatic predictions. Comparison of RNA sequencing based operon determination and bioinformatic predictions from MicrobesOnline [12,13]. **(e) **Distribution of mRNA lengths. The median mRNA length is 1,320 nucleotides, approximately twice the median size of an ORF (776 nucleotides) in S*. elongatus*.

One of the benefits of RNA sequencing is the ability to infer absolute mRNA transcript levels (Figure 2a). We calculated the absolute expression of each mRNA per cell, assuming a total of 1,500 mRNAs per cell [8,9] (Materials and methods; Table S1 in Additional file 1). We find that using this estimate, over 80% of mRNA transcripts are present at fewer than one copy per cell, suggesting an enormous diversity in single-cell transcriptome profiles and the potential for stochastic effects to play a substantial role in bacterial gene expression. Even if the estimated number of mRNAs per cell is four times larger (6,000 per cell), still nearly half (46%) of mRNAs are present at less than one copy per cell. Although an enormous amount of diversity in mRNA exists in each cell at any given time, the relatively rapid mRNA decay rates in cyanobacteria [10] - median 2.4 minutes in *Prochlorococcus *MED 4 - allow for rapid transcriptome turnover. The distribution of mRNAs per cell appears approximately log-normal with a dynamic range of almost 10,000. Most mRNAs fall within a smaller dynamic range of approximately 100, with a tail of higher expressed transcripts. The bottom part of the distribution was cut at 2^-4 ^because transcripts below this level are almost undetectable at our sequencing coverage (Materials and methods). The highest expressed KEGG (Kyoto Encyclopedia of Genes and Genomes) [11] categories include photosynthesis, ribosome, and RNA polymerase, with *P*-values of 2.6e-20, 1.3e-20, and 0.001, respectively (two-sided Wilcoxon rank sum test). The lowest expressed KEGG categories include mismatch repair, homologous recombination, and nucleotide excision repair - ORFs that may not be expressed in standard growth conditions (all *P *< 0.002, two-sided Wilcoxon rank sum test). Absolute transcript levels are generally correlated (Pearson correlation, r = 0.65) with RNA pol occupancy (Figure 2b), suggesting that transcription and not decay is the primary determinant for setting absolute transcript abundance. The variation (approximately one order of magnitude scatter) observed is roughly proportional to the expected distribution of mRNA decay rates in cyanobacteria [10]. However, this variation may also arise from: (1) different RNA pol elongation rates for different transcripts; (2) variable amounts of RNA pol pausing for different transcripts; and/or (3) lack of strand-specific information in the RNA pol ChIP data.

Of the 1,415 mRNA transcripts identified, many (approximately 38%) have more than one ORF per transcript (Figure 2c). Most mRNAs contain only one or two ORFs, but the ribosomal protein operon presents an extreme case of 31 ORFs on a transcript spanning over 17,000 nucleotides. Our operon identification via RNA sequencing shows good correlation with bioinformatic operon predictions from MicrobesOnline [12,13] (Figure 2d), which are based on: (1) distance between ORFs; (2) conservation of synteny in other genomes; and (3) commonality of Gene Ontology or COG category. The relatively high correspondence between RNA sequencing and bioinformatic predictions suggests that the operon structure in *S. elongatus *may be used to infer the operon structure in other cyanobacterial genomes. The median operon size is 1,320 nucleotides (Figure 2e), approximately twice the median size of an ORF (776 nucleotides) in *S. elongatus*.

### Transcription start

**Figure 3:**
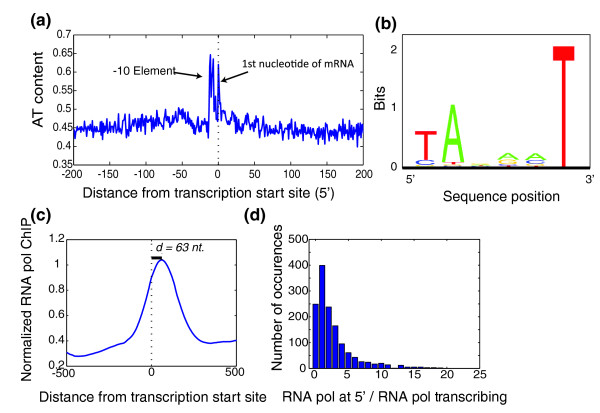
**Transcription initiation in *S. elongatus***. **(a) **AT content of the transcription start. The AT content from -200 to +200 from the start site of transcription was averaged for all mRNAs. A strong enrichment in AT content is observed at the -10 element as well as a strong preference for adenine at the first nucleotide of a transcript. **(b) **-10 element consensus logo. A consensus -10 element similar in sequence to that determined for *E. coli *was identified through sequence alignment (Materials and methods). **(c) **Normalized RNA pol occupancy at promoter. For each of the top 500 expressed mRNAs, the RNA pol occupancy was normalized to a mean occupancy of 0.5 per nucleotide, and then averaged across mRNAs from -500 to +500. A peak in RNA pol occupancy is observed, on average, 63 nucleotides within the RNA transcript, suggesting potential stalling of RNA pol after initiation of transcription rather than at the promoter. **(d) **RNA pol retention ratio at the promoter is variable. The relative amount of RNA pol at the 5' end versus RNA pol in the ORF varies from transcript to transcript. RNA pol at the 5' end was calculated as the mean occupancy in a 200-nucleotide window centered at the +63 nucleotide. RNA pol transcribing was calculated as the mean occupancy in a 200-nucleotide window centered in the middle of the first ORF.

Identification of the 5' ends of all mRNAs allows for more detailed characterization of the promoter and initial steps in transcription. When we align all mRNAs by their 5' transcription start and average their AT content, we observe an increase at the -10 element, also known as the Pribnow box [14,15] (Figure 3a). At this same location we observe a large drop in DNA melting temperature, a signature of bacterial promoters (Figure S4a in Additional file 2). Downstream of the -10 element, we detect a peak in AT content at the first nucleotide of the transcript, indicative of a preference for incorporating adenine (Figure S4b,c in Additional file 2). We computed the sequence alignment of the 30 nucleotides prior to the transcription start and find a -10 element similar to that found in a genome-wide map of transcription start sites in *Synechocystis *PCC 6803 and 25 experimentally determined promoters in *Prochlorococcus *MED4 [16-18] (Figure 3b; Materials and methods). Sequence alignment or motif analysis at the expected location of the -35 element or spacer does not reveal a strong consensus or motif. The absence of a strong -35 element signature has been observed in *Prochlorococcus *MED4 and in the *psbA *transcripts of many cyanobacteria [16,19], suggesting that the -35 elements in cyanobacteria may be very diverse in sequence. This diversity in -35 element may be related to the extensive control of gene expression by sigma factors in cyanobacteria [20].

To investigate the presence of RNA pol peaks near the transcription start site, we aligned the top 500 expressed transcripts by their 5' ends, and averaged the normalized RNA pol occupancy profiles (Figure 3c). On average, the maximum of the RNA pol peak is situated 63 nucleotides downstream of the transcription start site. The exact peak position varies from transcript to transcript; this peak can be located either in the 5' UTR or within the first ORF, with the majority of peaks occurring at the beginning of the ORF. This is in stark contrast to previous bacterial RNA pol ChIP-chip studies in which the RNA pol peaks are observed at the promoter [21-24], possibly due to a lack of resolution. A more recent high-resolution RNA pol ChIP-chip study in *Escherichia coli *was able to localize these RNA pol peaks to within the transcript [25].

To assess a potential functional role for these RNA pol peaks, we calculated the RNA pol retention as the ratio of RNA pol occupancy at the 5' end to the RNA pol occupancy in the middle of the first ORF for all mRNAs. We find that over 80% of transcripts have a retention ratio greater than one and that this retention ratio is variable from transcript to transcript (Figure 3d), allowing for the possibility that bacteria can tune the amount of retained RNA pol to affect gene expression.

One possible explanation for these RNA pol peaks is RNA pol pausing due to RNA secondary structure in nascent transcribed RNA, which may cause the RNA pol to pause or pause and subsequently terminate [26]. To determine if RNA secondary structure may be involved in pausing RNA pol, we selected a subset of 183 RNA pol peaks that were located 100 to 300 nucleotides within the transcript and were closer to a 5' end than a 3' end. This subset was chosen to specifically isolate the RNA pol peaks from features at the promoter or terminus, which may bias the analysis. The minimum free energy of 60-nucleotide RNA fragments from the transcribed strand, in 10-nucleotide increments, was calculated around each RNA pol peak and averaged, revealing a steep drop in the minimum free energy slightly prior to the RNA pol peak (Figure S5a in Additional file 2; Materials and methods). However, this decrease in free energy is still observed in dinucleotide shuffled sequences, suggesting that a specific stem loop structure is not formed in this region. Instead, we observe a shift in sequence bias from low to high GC content at the RNA pol peak (Figure S5b in Additional file 2), which may be influencing the RNA minimum free energy calculation. Thus, the mechanism underlying the global accumulation of RNA pol at the 5' end of transcripts remains unclear.

According to this hypothesis of 5' proximal RNA pol pausing, we should also observe enrichment of RNA sequencing reads at the 5' end of transcripts. Indeed, over 80% of transcripts have more RNA sequencing reads recovered at their 5' ends than in the middle of their first ORF (Figure S6a in Additional file 2), and a small but significant correlation exists between enrichment in RNA sequencing at 5' ends and RNA pol retention ratio (Figure S6b in Additional file 2).

Our genome-wide observations of 5' RNA pol peaks suggest that this may be a more important and widespread phenomenon in bacterial gene expression than previously appreciated. Our observations of RNA pol pausing may be different from the canonical examples of transcriptional attenuation observed in amino acid biosynthetic operons of *E. coli *where specific terminator structures attenuate transcription [26], although the peaks in RNA pol we observe are qualitatively similar to the peaks at the *trp *and *pyrBI *operons observed by tiling microarray in *E. coli *[25].

### Transcription termination

In addition to analysis of the transcription start, our catalog of 3' ends allows analysis of transcription termination. Two signals for transcription termination have been previously identified in bacteria: intrinsic Rho-independent terminators, typically low energy RNA hairpins; and Rho-dependent terminators, whose activity relies on the binding of the Rho protein to particular sites on the nascent transcript [27]. The majority of bacteria have a homolog of the *E. coli *Rho protein, but notable exceptions include the cyanobacteria *S. elongatus *and *Synechocystis *PCC 6803 [27]. A previous study analyzing the 3' ends of ORFs in *Synechocystis *PCC 6803 found no noticeable drop in RNA minimum free energy, suggesting the potential for a previously uncharacterized mechanism for transcription termination in this organism [28]. With knowledge of the actual 3' positions of transcripts, a more accurate analysis of transcription termination in *S. elongatus *is possible.

**Figure 4:**
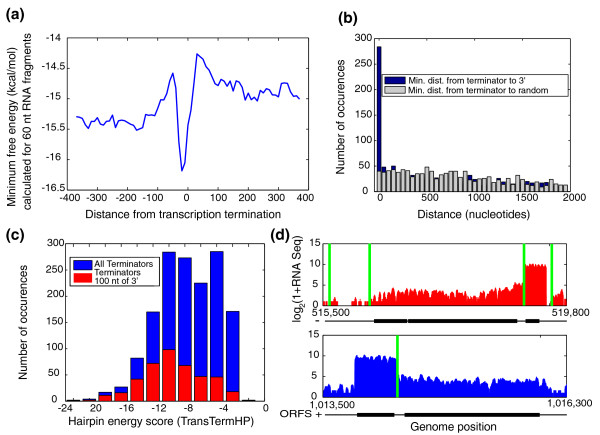
**Transcription termination in *S. elongatus***. **(a) **Minimum RNA free energy at the end of transcripts. The minimum free energy of 60-nucleotide RNA fragments with 10-nucleotide spacing was calculated and averaged for all mRNAs (Materials and methods). A drop in minimum free energy at the 3' end is indicative of Rho-independent transcription termination. **(b) **Distance between TransTermHP bioinformatically predicted terminators and 3' ends. Predicted intrinsic terminators (from TransTermHP [29]) tend to be much closer to the 3' end of transcripts than to random positions occurring at the same frequency as 3' ends. Blue bars show distance from a predicted terminator to the closest 3' end. As a control, we randomized the location of 3' ends in the genome. Grey bars show distance from a predicted terminator to the closest randomized 3' end. **(c) **Energy distributions of TransTermHP terminators. Not all predicted TransTermHP terminators cause transcription termination. Several terminator-like structures are located in non-transcribed regions or in the middle of transcripts. The free energy of terminators that cause transcription termination tends to be lower than the free energy of those that do not. The *P*-value is 3.00e-20 by two-sided Wilcoxon rank sum test. **(d) **Partial transcription termination creates complex transcriptional structures. Positive strand transcription is shown in blue and negative strand transcription in red. The positions of predicted terminators (from TransTermHP) are shown in green, and the position of JGI predicted ORFs are shown in black. Terminators located within transcripts often result in a decrease in the transcription of downstream ORFs.

To analyze the secondary structure at the 3' end of transcripts, we averaged the minimum free energy of all transcripts aligned by the 3' end (Figure 4a). We observe a dip in minimum free energy slightly prior to the transcript terminus, indicative of a stem-loop structure involved in Rho-independent transcription termination. This dip in free energy is not present in dinucleotide shuffled sequences, suggesting that a discrete stem-loop structure exists at the end of transcripts (Materials and methods; Figure S5c in Additional file 2). To further assess the role of Rho-independent transcription termination in *S. elongatus*, we assembled all Rho-independent intrinsic terminators predicted in *S. elongatus *from TransTermHP [29]. These predicted Rho-independent intrinsic terminators typically consist of short, often GC-rich hairpins followed by sequence enriched in thymine nucleotides. We find these terminators tend to be significantly closer to 3' ends than to random locations distributed at the same frequency (Figure 4b). Together, these analyses suggest that the classical Rho-independent termination plays a large role in cyanobacterial transcription termination.

Not all of the predicted intrinsic terminators cause complete transcription termination. The hairpin energy score (as calculated by TransTermHP [29]) of those terminators that are within 100 nucleotides of a transcription terminus tend to be lower (more negative) than those that are located elsewhere (Figure 4c). These more stable hairpins may be more competent to cause transcription termination because they are either more likely to fold and/or more likely to cause termination after folding [30]. In some cases, terminators that do not cause complete termination are involved in creating complex transcription structures. In several of these cases, terminators are found in between ORFs in the same operon, leading to lower transcription of the ORFs proximal to the 3' end (Figure 4d). This strategy could potentially be used to regulate the stoichiometry of transcript abundance of ORFs, and subsequently proteins, regardless of the state of the promoter. A potential physiological example is that of the phycocyanin operon where a terminator that causes incomplete termination sets the stoichiometry of mRNA for *cpcβ *and *cpcα *to phycobilisome rod linkers at 6:1 - the same stoichiometry as in the organized phycobilosome [31] (Figure S7 in Additional file 2).

### Putative non-coding transcripts and 5' UTRs

One particularly interesting feature of the *S. elongatus *transcriptome is the presence of widespread non-coding transcription. We identify 1,579 putative non-coding transcripts from RNA sequencing, 983 of which are considered high-confidence after verification by tiling microarray, and annotate their 5' and 3' ends (Table S2 in Additional file 1; Materials and methods). The number of non-coding transcripts is comparable to the number of annotated protein-coding transcripts (1,415). It is possible that some of the transcripts designated as non-coding may have a protein coding region that was not identified in the JGI annotation. Those putative non-coding transcripts that have any overlap with annotated transcripts on the opposite strand were considered anti-sense and the remaining were considered not anti-sense.

Several hundred non-coding RNAs have previously been identified in *E. coli *and *Bacillus subtilis *[32] and recently 276 novel transcriptional units were identified in *Prochlorococcus *MED4 by tiling microarray [33], 117 in *Mycoplasma pneumonia *by tiling microarray and transcriptome sequencing [34], 390 in *Sulfolobus solfataricus *P2 by transcriptome sequencing [35], and 137 in *Salmonella *Typhi by transcriptome sequencing [36]. As RNA from these and other genomes are sequenced at further depth, we may find that non-coding transcription is more prevalent in bacteria than previously thought [37-39]. A recent RNA sequencing-based map of transcription start sites in another unicellular cyanobacterium, *Synechocystis *PCC 6803, identified 1,541 potential non-coding transcription start sites, making up 64% of all transcription start sites in the organism [17].

We find that some of the non-coding transcripts in *S. elongatus *display differential expression in the subjective dawn and subjective dusk timepoints, indicative of circadian expression, as assayed by tiling microarray (Table S2 in Additional file 1, Figure S8 in Additional file 2; Materials and methods). Although several non-coding RNAs appear to exhibit circadian oscillations in expression, the physiological role for circadian gene expression remains unclear and no expression correlation exists between anti-sense circadian non-coding RNAs and the transcripts on the opposite strand.

**Figure 5:**
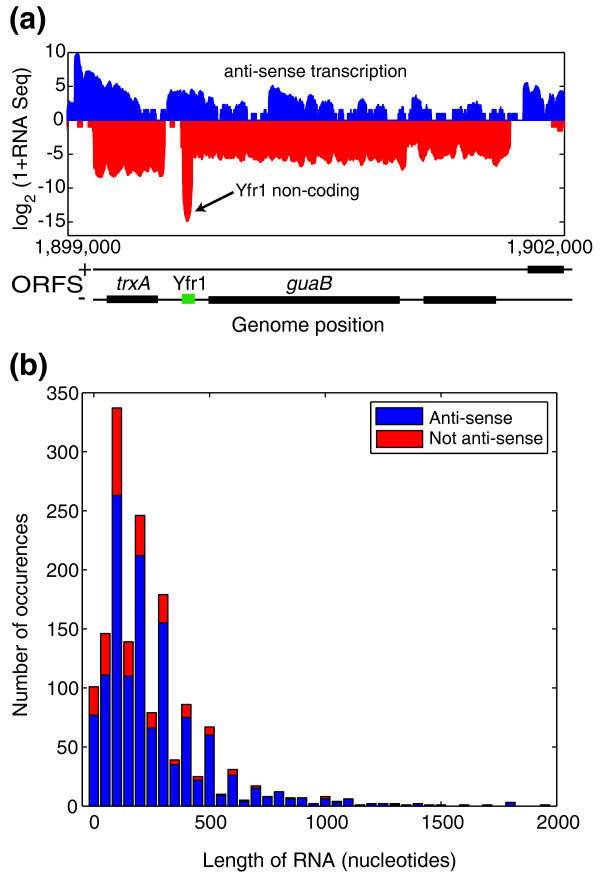
**Non-coding transcripts**. **(a) **Extensive non-coding transcription. Positive strand transcription is shown in blue (positive y-axis), and negative strand transcription in red (negative y-axis). The position of JGI predicted ORFs on the plus and minus strand are shown in black, and the position of the Yfr1 non-coding RNA is shown in green. In this same region, there is anti-sense transcription opposite to the *trxA *and *guaB *ORFs. **(b) **Length distribution of non-coding transcripts. Transcripts that have any overlap with an annotated transcript on the opposite strand are designated anti-sense. Most non-coding transcripts are anti-sense by this designation. The median size for a non-coding transcript is approximately 200 nucleotides.

Very few well-described examples of non-coding RNAs have been noted in cyanobacteria. One previously identified functional non-coding RNA, Yfr1, is required for growth under several stress conditions [40] (Figure 5a). In *S. elongatus*, there appears to be occasional co-transcription of Yfr1 with the neighboring ORF *guaB*, but the extent of co-transcription is negligible compared to the expression of Yfr1. In the same genomic region as Yfr1, we observe several previously unidentified transcripts anti-sense to the *trxA *and *guaB *coding regions. The Yfr1 non-coding transcript is approximately 60 nucleotides in length, and the median size of all identified non-coding transcripts is approximately 200 nucleotides, roughly 15% of the size of mRNA transcripts (Figure 5b).

We find that most non-coding transcripts are at least partially anti-sense to an mRNA transcript (Figure 5b). These transcripts have the potential for base pairing with the transcript on the opposite strand. One such functional RNA, IsrR, has been identified in the cyanobacterium *Synechocystis *PCC 6803 [41]. This 177-nucleotide RNA is down-regulated in iron stress and base-pairs with the iron stress-induced *isiA *transcript, subsequently decreasing its levels. IsiA enhances photosynthesis by forming a ring around photosystem I, and IsrR is currently the only RNA known to regulate a photosynthesis component [41]. We find a transcript anti-sense to *isiA *in *S. elongatus *that shows significant similarity to IsrR in *Synechocystis *PCC 6803 (RNA Families (RFAM) bit score 97.96) [42]. This transcript may have a similar role in modulating photosynthesis in *S. elongatus*.

To identify if any other known RNA families are present within our set of non-coding RNAs, we queried the RFAM database [42]. In addition to Yfr1, IsrR, and RNase P, we identify a non-coding RNA containing a putative group I intron [43]. Group I introns are ribozymes capable of catalyzing their own excision from an RNA, and ligating the upstream and downstream exons.

To extend our analysis of potential RNA-based regulators in *S. elongatus*, we queried our set of 5' UTRs against RFAM and identified metabolite-binding riboswitches for thiamine (vitamin B_1_) and coenzyme B_12 _(vitamin B_12_). The 5' leader of the *thiC *mRNA in *S. elongatus *contains a 'thi box' riboswitch domain that undergoes a structural change that has been shown to cause both a reduction in translation and transcription when bound to thiamine or its pyrophosphate derivative [44]. Similarly, the 5' leader of a putative cobalt transporter (JGI 637799805, *Synpcc7942_1373*) contains the cobalamin riboswitch domain, which represses expression in the presence of coenzyme B_12 _[45]. Both of these mRNA transcripts have unusually large 5' UTRs of 210 and 153 nucleotides, respectively, compared to a median 5' UTR size in *S. elongatus *of 30 nucleotides. Although most 5' UTRs are small, 12% are longer than 100 nucleotides and 6% are longer than 150 nucleotides. Transcripts with long 5' UTRs may be good candidates for riboswitches or RNA-based regulators. Interestingly, both riboswitch-containing mRNAs show large RNA pol occupancy peaks near the riboswitch domain in the 5' UTR, suggesting that these riboswitches - likely when in their bound configuration - can cause RNA pol pausing or termination. These peaks in RNA pol are qualitatively similar to the peaks we observe globally, although mechanisms likely differ, as most RNA pol peaks are situated within the beginning of the ORF.

## Conclusions

Here we combine three high-resolution data sets - RNA sequencing, tiling expression microarray, and RNA pol ChIP sequencing - to present a characterization and analysis of the *S. elongatus *transcriptome. We report absolute transcript levels, operon identification, and high-resolution mapping of 5' and 3' transcript ends. At the 5' end of transcripts, we characterize promoter sequence and find widespread peaks in RNA pol occupancy. At 3' ends we observe significant Rho-independent transcription termination and occasional incomplete termination resulting in interesting transcriptional structures. In addition, we find extensive non-coding transcription, suggesting a larger role for these non-coding RNAs in bacteria, and cyanobacteria in particular, than previously anticipated. The presence of numerous non-coding RNAs and 5' proximal pausing of RNA pol suggest that post-transcriptional regulation - regulation after binding of RNA pol at the promoter - may be more widespread in bacteria than expected. We hope this work will serve as a catalog and primer for further studies of bacterial and cyanobacterial transcription.

## Materials and methods

### Continuous culture of cyanobacteria

Cyanobacteria were cultured as previously described [3]. A continuous culture apparatus kept cells in constant light and growth conditions and provided real-time bioluminescence readings. *S. elongatus *(stain AMC 408 [46]): *psbAI::luxCDE *fusion in NS1 [47] (spectinomycin and streptomycin) and *purF::luxAB *fusion in NSII [47] (chloramphenicol) was grown in a 6-L cylindrical spinner flask (Corning, Corning, NY, USA) at a volume of 4.5 L. Cells were grown in BG-11 medium [48] with the following modifications: 0.0010 g/L FeNH_4 _citrate was used instead of 0.0012 g/L FeNH_4 _citrate and citric acid was supplemented at 0.00066 g/L. Cells were initially inoculated in the presence of antibiotics (5 μg/ml spectinomycin and 5 μg/ml chloramphenicol), and subsequently diluted with modified BG-11 lacking antibiotics. Cells were exposed to surface flux of approximately 25 μmol photons m^-2 ^s^-1 ^cool white florescent light, bubbled with 500 ml/minute 1% CO_2 _in air, maintained at 30°C, and stirred at one rotation per second. Constant optical density (OD_750 _0.15) and volume are achieved via a two state controller. OD does not fluctuate greater than 8% during an experiment. Cells are exposed to two 12-hour light-dark cycles for entrainment before release into continuous light.

### RNA preparation

Total RNA was prepared as previously described [3]. Cells (120 ml) from continuous culture were collected by vacuum filtration, snap frozen in liquid nitrogen, and stored at -80°C for no more than 1 week prior to RNA extraction. RNA was extracted from frozen cells in two steps. First, cells were lysed in 65°C phenol/SDS by vortexing and total RNA was purified by phenol/chloroform extraction. Second, total RNA was subjected to DNase I (Promega, Madison, WI, USA) treatment followed by a second phenol/chloroform extraction. Total RNA was analyzed on agarose gel and an Agilent Bioanalyzer to assess integrity.

### Strand-specific RNA sequencing

Total RNA was prepared for timepoints collected at 4-hour intervals from 76 to 96 hours after release into continuous light and mixed in equal proportions. Mixed total RNA was supplemented with RNase Out (Invitrogen, Carlsbad, CA, USA) to a final concentration of 2 units/μl and depleted of 23S and 16S ribosomal subunits using the MICROBExpress Bacterial mRNA Enrichment Kit (Ambion, Austin, TX, USA) according to manufacturer's instructions.

RNA sequencing libraries were prepared from total RNA depleted of 16S and 23S rRNA with modifications to a previously described procedure [5]. RNA (8 μg) was fragmented for 40 minutes at 95°C in fresh 2 mM EDTA, 100 mM NaCO_2_, pH 9.2. Fragmentation reactions were immediately precipitated in 300 mM NaOAc, pH 5.2, glycogen, and isopropanol. Fragmented RNA was resuspended in RNA loading buffer (Fisher, Pittsburg, PA, USA), briefly denatured, and loaded in a 15% TBE-Urea polyacrylamide gel (BioRad, Hercules, CA, USA) for size selection. Gels were stained with Sybr Gold (Invitrogen) and a 25- to 30-nucleotide band was excised using a synthesized 28-nucleotide RNA and denatured 10-bp DNA ladder (Invitrogen) as standards. The gel slice was physically disrupted and RNA was recovered in 300 mM NaOAc, 1 mM EDTA, 0.1 units/μl SUPERase·In (Ambion) overnight at room temperature. Solution was transferred to a Spin-X cellulose acetate filter (Corning) to remove gel debris and precipitated with glycogen and isopropanol. Size selected fragmented RNA was denatured briefly and dephosphorylated in a 30 μl reaction with 1 × T4 polynucleotide kinase buffer without ATP (NEB, Ipswich, MA, USA), 20 units SUPERase·In, and 15 units T4 polynucleotide kinase (NEB) at 37°C for 1 hour. The reaction was precipitated, resuspended, briefly denatured, and poly-(A) tailed in a 25 μl reaction with 1 × poly-(A) polymerase buffer (NEB), 5 units SUPERase·In, 1 mM ATP, and 1.25 units *E. coli *poly-(A) polymerase (NEB) at 37°C for 10 minutes. Reactions were quenched with 80 μl of 5 mM EDTA and precipitated.

Reverse transcription was carried out from the introduced poly-(A) tail anchor of denatured RNA using primer oNTI255 [5] with the SuperScript III reverse-transcriptase system (Invitrogen) supplemented with 2 units/μl of SUPERase·In at 48°C for 30 minutes. RNA was subsequently hydrolyzed in 0.1 M NaOH at 98°C for 15 minutes and loaded in a 10% TBE-Urea polyacrylamide gel (BioRad) and the extended first-strand product was excised and recovered as above in 300 mM NaCl, 10 mM Tris, pH 7.9, 1 mM EDTA. First-strand cDNA was circularized in a 20 μl reaction with 1 × CircLigase buffer (Epientre, Madison, WI, USA), 50 μM ATP, 2.5 mM MnCl_2_, and 1 μl CircLigase (Epientre) for 1 hour at 60°C, and then heat-inactivated for 10 minutes at 80°C.

Circularized cDNA template (1 μl) was amplified using Phusion Hot Start High-Fidelity enzyme (NEB) and primers oNTI230 and oNTI231 [5] to create DNA with Illumina cluster generation sequences on each end along with the Illumina small RNA sequencing primer binding site. PCR was carried out with an initial 30 second denaturation at 98°C, followed by 8 cycles of 10 second denaturation at 98°C, 10 second annealing at 60°C, and 5 second extension at 72°C. PCR product was loaded in a non-denaturing 10% TBE polyacrylamide gel (BioRad) and a 113- to 125-nucleotide band was excised using a 10-bp ladder as standard. DNA was recovered as previously described. Libraries were quantified using an Agilent Bioanalyzer and 4 to 6 pM of template was used for cluster generation and sequenced on Illumina Genome analyzer II with the Illumina small RNA sequencing primer. Sequence tags were stripped of the terminal poly-(A) sequence and aligned to the *S. elongatus *genome with Bowtie [49]. Stripping of terminal poly-(A) sequence at the end of each read will remove the introduced poly-(A) tail but will also remove any trailing adenines at the 3' end of the reverse-transcribed RNA fragment, biasing the 3' end determination of RNAs that end in trailing adenines. GenBank CP000100, CP000101, and S89470 were used to align reads to the chromosome and endogenous plasmids. Uniquely mappable reads with a maximum of three mismatches were mapped to the genome and extended by the length of the individual read.

A total of 22,375,035 uniquely mappable reads were mapped to the genome with approximately 624 million bases of sequences covering each nucleotide strand-specifically an average of approximately 115 times. These uniquely mappable reads exclude any reads from rRNA since multiple copies of each rRNA exist in the genome. Technical replicates showed very high Pearson correlation coefficients (r > 0.99). RNA sequencing data are displayed and analyzed as coverage per nucleotide - defined as the number of times a given nucleotide position was observed in all the sequencing reads. Absolute transcript levels are assumed to be equal to the average coverage per nucleotide across the length of the transcript. All analysis was performed on the chromosome, although raw data for both endogenous plasmids are available.

### Strand-specific expression tiling microarray

Expression was measured using two separate custom designed two-color 244 k microarrays - one for the forward strand and another for the reverse strand (forward strand tiling array, Agilent Array ID 022715; reverse strand tiling array, Agilent Array ID 022716). Arrays were designed using eArray software (Agilent). Forward and reverse strand sequence is as defined by GenBank CP000100, CP000101, and S89470 - which define the chromosome and two plasmid sequences, respectively.

All tiling probes were 60 nucleotides in length with 12-nucleotide spacing between probe starts such that probe_i _and probe_i+1 _overlapped by 48 nucleotides. A 6-nucleotide offset of the tile between strands allows for 6-nucleotide resolution of double stranded targets and 12-nucleotide resolution for strand-specific targets. In addition, each array included four temperature matched probes (80°C) against each JGI predicted ORF, *luxA *through *luxE*, and *Arabidopsis *spike-in controls (Ambion). These additional probes are identical to those in Agilent Array ID 020846, as previously described [3].

cDNA was prepared for each individual timepoint (foreground channel) as well as for a pool of all timepoints (background channel). The background channel consisted of a pool of samples collected at 4-hour intervals from 24 to 84 hours after release into continuous light. The foreground channel consisted of individual timepoints 60, 68, 72, and 80 hours after release into continuous light. The same samples were analyzed by non-tiling microarray in [3]. Spike-in RNA was introduced at different concentrations and ratios to the foreground and background channels before reverse transcription to ensure proper ratio detection over a wide dynamic range. Total RNA (5 μg; plus spike-ins) was reverse-transcribed with random 15-mer primers (Operon, Huntsville, AL, USA) and a 2:3 ratio of amino allyl-UTP:dTTP (Sigma, St. Louis, MO, USA) using the SuperScript III reverse-transcriptase system without amplification. RNA was hydrolyzed and cDNA was purified using Microcon 30 spin column (Millipore, Billerica, MA, USA).

First-strand cDNA was labeled with *N*-hydroxysuccinimide-ester cyanine 3 (Cy3, foreground) or cyanine 5 (Cy5, background) (GE Biosciences, Uppsala, Sweden) in 0.1 M sodium bicarbonate pH 9.0 for 6 hours. Labeled cDNA was purified (Microcon 30) in preparation for hybridization. Each array was hybridized with approximately 750 ng Cy3 and approximately 750 ng Cy5 labeled cDNA and rotated (five rotations per minute) at 60°C for 17 hours in SureHyb chambers (Agilent). Arrays were subsequently washed in 6.7 × SSPE and 0.005% N-lauryl sarcosine buffer for at least 1 minute, 0.67 × SSPE and 0.005% N-lauryl sarcosine buffer for 1 minute, and then Agilent drying and ozone protection wash for 30 seconds at room temperature (1 × SSPE = 0.15 M NaCl, 10 μM sodium phosphate, 1 mM EDTA, pH 7.4). The arrays were immediately scanned using an Axon 4000B scanner at 5-μm resolution. The median intensity of the Cy3 and Cy5 florescence at each spot was extracted using GenePix software (Molecular Devices, Sunnyvale, CA, USA). For calculation of logarithmic ratios, Loess and quantile normalization were performed in succession using the MATLAB (MathWorks, Natick, MA, USA) bioinformatics toolbox.

### ChIP sequencing of RNA polymerase

We crosslinked 250 ml of cells from continuous culture (OD_750 _0.15) with 1% formaldehyde for 15 minutes and then quenched them with 125 mM glycine for 5 minutes at room temperature. Cells were collected by centrifugation and washed twice with cold phosphate-buffered saline buffer, pH 7.4. The cell pellet was snap frozen in liquid nitrogen and stored at -80°C. Samples were collected 32 to 52 hours after release into continuous light at 4-hour intervals. At the same time, samples were collected and processed for non-tiling microarray as described in [3].

ChIP was performed in a manner similar to that previously described [50,51]. Cells were mechanically lysed by beating with 0.1 mm glass beads in cold lysis buffer A (50 mM HEPES, pH 7.5, 140 mM NaCl, 1 mM EDTA, 1% Triton X-100, 0.1% Na-Deoxycholate) with protease inhibitors (Roche, Basel, Switzerland). Chromatin was fragmented by sonication of the lysate to a median of approximately 300 bp and the protein concentration of the supernatant was measured by BCA (bicinchoninic acid) (Thermo, Rockford, IL, USA) using bovine serum albumin as standard. Lysate (750 μg) was incubated with 30 μg of antibody - RNA polymerase β subunit antibody WP023 (Neoclone, Madison, WI, USA) or mouse whole IgG mock (Jackson ImmunoResearch, West Grove, PA, USA) - and incubated overnight at 4°C. We verified that the monoclonal RNA polymerase β subunit antibody WP023 reacts with *S. elongatus *RNA polymerase β by western blot analysis of whole cell extract, where it produces a single band of the expected size. Lysate was supplemented with Protein G Sepharose Fast-Flow beads (Invitrogen) and incubated for an additional 2 hours at 4°C. After incubation, sepharose beads were washed in cold buffer at room temperature: 2 × 5 minutes lysis buffer A; 1 × 5 minutes lysis buffer B (50 mM HEPES, pH 7.5, 500 mM NaCl, 1 mM EDTA, 1% Triton X-100, 0.1% Na-deoxycholate); 1 × 5 minutes wash buffer (10 mM Tris-HCl, pH 8.0, 250 mM LiCl, 1 mM EDTA, 0.5% NP-40, 0.1% Na-deoxycholate); 1 × 5 minutes TE buffer (10 mM Tris-HCl, pH 8.0, 1 mM EDTA). Protein-DNA was eluted from beads by incubation of samples at 65°C for 1 hour in elution buffer (50 mM Tris-HCl, pH 8.0, 10 mM EDTA, 1.0% SDS). Crosslinks were reversed in supernatant by incubation of samples at 65°C overnight in elution buffer. Western blotting of supernatant of mock versus immunoprecipitation shows 45% efficiency pull-down of the β subunit and 25% co- immunoprecipitation of the β' subunit in the immunoprecipitation using Neoclone antibodies WP023 and WP001, respectively. Proteins were digested with 0.2 mg/ml proteinase K for 2 hours at 37°C. Nucleic acid was then purified with phenol/chloroform extraction and precipitated with ethanol and LiCl. Nucleic acid was re-suspended in TE buffer and RNA was digested in 20 μg/ml RNase and subsequently phenol/chloroform purified. For input control, 5% of the volume of cell lysate was removed after sonication and used to prepare the input DNA. The ChIP DNA concentration was estimated with the Pico-green DNA detection kit (Invitrogen).

ChIP sequencing libraries were prepared for samples zeitgeber time (ZT) 32 (subjective dusk) and ZT 44 (subjective dawn) as these timepoints showed maximal/minimal gene expression for canonical circadian mRNAs *kaiC *and *purF *by microarray. Mock ChIP sequencing libraries were prepared for an equal mix of lysate from ZT 32 through ZT 52 (collected at 4 hour intervals). A total of six sequencing libraries were prepared (Table 1).

**Table 1:** RNA polymerase ChIP samples

Sample	Total aligned reads
ZT 32 RNA pol ChIP	8,815,678
ZT 32 input	20,203,310
ZT 44 RNA pol ChIP	11,201,620
ZT 44 input	19,864,425
ZT 32 through 52 mock	10,595,684
ZT 32 through 52 input	16,712,868

ZT, zeitgeber time.

Sequencing libraries were prepared from 10 ng DNA following the Illumina ChIP protocol (revision A) and libraries sized between 200 and 300 bp were selected for amplification. Libraries were assayed with the Agilent Bioanalyzer and 8 pM of template was used for cluster generation. Libraries were sequenced using Illumina primers on an Illumina Genome analyzer II, and each sequence tag was aligned to the *S. elongatus *genome with Bowtie [49]. GenBank CP000100, CP000101, and S89470 were used to align reads to the chromosome and endogenous plasmids. Uniquely mappable reads with a maximum of three mismatches were mapped to the genome. Reads were then extended 150 bp to cover the average length of insert DNA between sequencing adaptors as determined by the Agilent Bioanalyzer.

A comparison of change in RNA pol ChIP versus change in gene expression (measured by non-tiling microarray) at timepoints ZT 32 and ZT 44 is shown in Figure S3 in Additional file 2. All other analysis was performed on the sum of the normalized libraries from ZT 32 and ZT 44, which was normalized to a mean coverage of 200 reads per nucleotide. Additional normalization by input does not change conclusions (Figure S2 in Additional file 2). A representative region of the genome is presented in Figure S2 in Additional file 2. All analysis was performed on the chromosome, although raw data for both endogenous plasmids is available.

### Calculation of percent of genome transcribed

The percent of transcription along the *S. elongatus *chromosome was calculated by imposing a coverage cutoff for transcription of two reads per nucleotide. If a nucleotide is expressed at or over this cutoff it is regarded as transcribed.

This conservative cutoff indicates that only approximately 84.7% of the nucleotides within annotated JGI chromosomal ORFs are transcribed. Of the approximately 15.3% of nucleotides within annotated ORFs that do not pass this cutoff, approximately 41.4% are within an ORF that has an average number of reads per nucleotide of less than 2, which corresponds to approximately 1 RNA per 15 cells when we assume a total of 1,500 mRNAs per cell (245 of 2,665 chromosomal ORFs have <2 reads per nucleotide).

Using this cutoff we find 54.7% of each strand is transcribed and 88.0% of the chromosome is transcribed on either the plus or minus strand. That is, on any given strand and at any given chromosomal position, there is a 54.7% chance that the nucleotide is transcribed. Similarly, at any given chromosomal position, there is an 88% chance that the nucleotide is transcribed on either the plus or minus strand. Eighty-two percent of non-coding regions are transcribed on either the plus or minus strand.

### Identification of 5' and 3' ends of Joint Genome Institute predicted ORFs and definition of operons

The 5' and 3' ends of all JGI predicted ORFs (and rRNA and tRNA) with an average coverage of at least two reads per nucleotide were identified using a probability-based approach using *a priori *knowledge of translation start and stop positions. Of 2,665 chromosomal ORFs (and rRNA and tRNA), 2,420 had an average number of reads per nucleotide of ≥2. For every predicted translation start, we searched for the first upstream nucleotide (*i *- 1 is upstream of *i*) on the same strand *i *that was not within a JGI predicted ORF and that satisfied one of the following three criteria: (1) binomial_cdf _( reads_*i-1*_, reads_*i *_+ reads_*i-1*_, 0.5 ) < 0.01 and reads_*i*_/reads_*i-1 *_≥ 2; (2) binomial_cdf _( reads_*i-2*_, reads_*i *_+ reads_*i-2*_, 0.5 ) < 0.01 and reads_*i*_/reads_*i-2 *_≥ 2; and (3) reads_*i-1 *__<_2.

Where binomial_cdf _(k, n, p) is the probability of getting at least k success in n trials when p is the success probability of each trial. This *i *was designated the 5' transcription start site. The distance of predicted 5' ends to those published in previous studies is reported in Table S4 in Additional file 1 and examples are shown in Figure S1 in Additional file 2. Similarly, for every predicted translation stop codon, we searched for the first downstream nucleotide *i *that was not within a JGI predicted ORF and that satisfied one of the same criteria. This *i *was designated the 3' transcription end. 5' Ends tend to be better defined than 3' ends, possibly related to the biology of transcription termination. ORFs that shared the same 5' transcription start site were defined as being on the same operon. We observed 43 cases of multiple transcription start sites - the presence of a 5' transcription start within another transcript. All identified transcripts are reported in Table S1 in Additional file 1. A total of 1,473 transcripts were identified. All analysis was performed on the subset of 1,415 transcripts defined as mRNA transcripts as they do not contain any tRNA or rRNA. Note, in some cases a tRNA was predicted to be on the same transcript as an ORF because the high expression of the tRNA obscures the transcription boundary.

### Identification of non-coding transcripts

Non-coding transcripts were identified using a multi-tiered approach that first identifies transcribed regions and then estimates their 3' and 5' positions.

First, 15,000 nucleotide intervals of the chromosome (with overlap of 5,000 nucleotides) were optimally segmented into 30 segments of approximately constant signal, yielding a total of 8,070 segments per strand. Segmentation was performed in MATLAB to minimize the cost function:

where *y*_*i *_is the log_2_(1 + reads_i_) at nucleotide *i*,  is the arithmetic mean of log_2_(1 + reads) along segment *s*, *t*_1_, ..., *t*_*s *_and are segment boundaries [52-54]. This change-point approach more accurately discriminates transcribed and non-transcribed segments than the running window approach and requires only one user-defined parameter - the total number of transcribed segments - which we set at 1 per 500 nucleotides strand-specifically.

Next, all segments that correspond to non-transcribed regions - mean coverage less than two reads per nucleotide - were removed. Segments that overlapped with an annotated transcript (see previous section) were removed and the remaining segments were consolidated. The exact 5' and 3' end of each segment was determined using the same algorithm described in the previous section except 5' and 3' ends were not allowed to overlap with an annotated operon. A total of 1,579 non-coding transcripts were detected using this method. All non-coding transcripts are reported in Table S2 in Additional file 1.

### Identification of high-confidence non-coding transcripts

Tiling microarray ratios were utilized to identify a set of high-confidence non-coding transcripts. We took advantage of the fact that transcripts have high Pearson cross-correlation among internal probes (probes that are fully internal to the transcript) across all circadian timepoints [55]. That is, when the ratio of one probe changes at a particular circadian time, the ratio of the other probes within the transcript is similarly affected. First, we assembled the distribution of mean cross-correlation values among internal probes for all predicted JGI ORFs. This formed the expected cumulative distribution for mean cross-correlation of transcribed regions. All non-coding transcripts whose mean cross-correlation was above the 5% cutoff of the expected distribution were considered high-confidence. This assumes that all non-coding transcripts with mean cross-correlation larger than the bottom 5% of ORFs are high-confidence. Table S2 in Additional file 1 indicates whether a non-coding transcript was designated as high-confidence. Of the 1,579 non-coding transcripts, 157 could not be assayed because they were smaller than the probe width of 60 nucleotides. Of the remaining 1,422 non-coding transcripts, 983 (approximately 70%) passed this cutoff.

### Identification of high-confidence circadian non-coding transcripts

Circadian transcripts corresponding to annotated JGI ORFs have been previously described [3,4]. To identify potential non-coding circadian transcripts, we first calculated the relative gene expression of each non-coding transcript at each timepoint by taking the arithmetic mean of gene expression ratios across all microarray probes internal to the transcript. This gives us the relative expression of each non-coding transcript at each timepoint relative to the background. Then we calculated the gene expression ratio between the two most extreme (in gene expression) circadian timepoints (circadian time (CT) 12 (subjective dusk) and CT 20 (subjective dawn), corresponding to ZT 60 and ZT 72, respectively) [3]. Large negative ratios are indicative of dawn-peaking transcripts and large positive ratios are indicative of dusk-peaking transcripts. To assign a designation of circadian behavior to each non-coding transcript, we calculated the same ratios for all annotated ORFs - where the circadian behavior is already known from [3]. We found the ratio for annotated ORFs at which a cumulative 10% false positive rate existed for dawn or dusk genes, and used these cutoffs to identify potential circadian non-coding transcripts. Expression ratios and indication of potential circadian behavior are shown in Table S2 in Additional file 1. The timecourse expression of all high-confidence circadian non-coding RNAs is shown in Figure S8 in Additional file 2. Although only 106 of 1,579 non-coding transcripts pass this strict cutoff (10% false-positive rate), by comparing the distribution of ratios for annotated and non-coding transcripts, we estimate that a total of 817 non-coding transcripts are circadian.

### Identification of RNA polymerase peaks

RNA pol ChIP peaks were identified in the sum of timepoints ZT 32 and ZT 44 hours using a maxgap/minrun approach similar to the first pass of PeakSeq [56]. All peaks larger than 100 nucleotides and separated by at least 20 nucleotides in the ChIP sample were assembled for thresholds starting from the mean coverage to ten times the mean coverage with increments of one-twentieth mean coverage. The unique peaks were selected and consolidated such that no peak maximums are within 150 nucleotides of each other. This method accurately captures the wide dynamic range of peaks present in the data. All RNA pol peaks and their enrichment over mock are reported in Table S3 in Additional file 1; 87% of RNA pol peaks are enriched over the mock (*P *< 0.1). Those peaks that are not enriched over mock appear to be actual peaks in RNA pol ChIP, but these RNA pol ChIP peaks are smaller than the mock background, which is elevated with respect to the ChIP background after both data sets are normalized for the number of reads (Figure S2 in Additional file 2). All RNA pol peaks were used in analysis and results do not change when only peaks enriched over the mock are used. Figure S2 in Additional file 2 shows peak identification over a representative genomic region.

### Distribution of mRNA per cell

The distribution of mRNA per cell was calculated by assuming a total of 1,500 mRNAs per cell [8,9]. For each mRNA species *m*_*1*_,..., *m*_*1415*_, the abundance of the species *m*_*i *_per cell was given by:

where *γ*_*i *_is the mean number of reads per nucleotide within the mRNA species *i*. All mRNA-per-cell estimates are reported in Table S1 in Additional file 1. Only mRNAs with *γ*_*i *_greater than 2 are shown in Figure 2a.

### Calculation of minimum free energy of secondary structure of RNA

Minimum free energy of secondary structure of RNA was calculated with MATLAB Bioinformatics Toolbox command *rnafold *- minimum free energy is calculated using a thermodynamic nearest-neighbor approach [57,58] and is reported in kcal/mol. All free energies are calculated on 60-nucleotide RNA fragments using a sliding window of 10 nucleotides.

To test whether minimum free energy changes were dependent on dinucleotide frequency of the RNA, dinucleotide shuffled sequences with the same overall dinucleotide content distribution were generated using a first order Markov model. That is, for each position in the sliding window, the dinucleotide content of all sequences was assembled. Then an equal number of dinucleotide shuffled sequences were randomly generated maintaining the same overall dinucleotide content distribution.

At the 3' end of transcripts, a dip in minimum free energy was not observed in the dinucleotide shuffled sequences, but was observed in native sequences (Figure S5c in Additional file 2). In addition, the minimum free energy at the dip in native sequences (mean = -16.11 kcal/mol) was significantly lower than that in dinucleotide shuffled sequences at the same position (mean = -13.95 kcal/mol; Z = -0.52, *P *= 1.66e-31). Z-scores were calculated as the difference in mean of native and dinucleotide shuffled sequences divided by the standard deviation of dinucleotide shuffled sequences and *P*-value was calculated using the two-sided Wilcoxon rank sum test. This suggests that a particular stem-loop feature, likely associated with transcription termination, is present at the end of transcripts.

At the RNA pol peaks at the 5' ends of genes (Figure S5a in Additional file 2), the change in minimum free energy in native and dinucleotide shuffled sequences was nearly identical, suggesting that changes in dinucleotide (or nucleotide) frequency and not a discrete stem loop structure are responsible for the transition in free energy. A change in nucleotide content does occur at the position of the RNA pol peaks (Figure S5b in Additional file 2), and may play a role in RNA pol pausing by an unknown mechanism. A drop in minimum free energy in native and dinucleotide shuffled sequences is also observed globally when all transcripts are aligned by their 5' end (Figure S5d in Additional file 2). A similar change in nucleotide content occurs approximately 100 nucleotides from the 5' end of transcripts (Figure S4b in Additional file 2). These global sequence changes proximal to the 5' end of transcripts may coincide with our observation of global RNA pol pausing internal to the 5' ends of transcripts.

### Calculation of DNA melting temperature

Melting temperature was calculated with MATLAB Bioinformatics Toolbox command *oligoprop *- melting temperatures are calculated using a nearest-neighbor approach with default parameters [59].

### Identification of -10 element in promoters

All unique mRNA transcription start sites were aligned and the +1 to -30 sequences were input into CONSENSUS-V6C [60], which finds a consensus pattern of defined width (width = 8 nucleotides) in unaligned sequences. This procedure identified 5' --Ta-aaT 3' motif, corresponding to the -10 element (Pribnow box), with ln(p) = -4092.23 where p is the probability of identifying a motif with the same or higher information content in an arbitrary alignment. This motif was found at slightly different positions in each of the sequences. To identify the true -10 element while removing any potential false positives, the motif from the subset of alignments that identified the initial nucleotide of the motif at -8 (285 of 1,416 transcripts) is shown in Figure 3b. In subsequent searches using CONSENSUS-V6C or other motif algorithms, no motif was found downstream of the -10 motif where a -35 motif may be expected.

## Abbreviations

bp: base pair; ChIP: chromatin immunoprecipitation; CT: circadian time; HIP1: highly iterated palindrome 1; JGI: Joint Genome Institute; OD: optical density; ORF: open reading frame; RFAM: RNA Families; RNA pol: RNA polymerase; UTR: untranslated region; ZT: zeitgeber time.

## Competing interests

The authors declare that they have no competing interests.

## Authors' contributions

VV and EKO designed experiments; VV and IHJ performed experiments; VV and IHJ analyzed data; VV and EKO wrote the paper. All authors have read and approved the manuscript.

## Data availability

All data sets have been uploaded to the Gene Expression omnibus under accession [GEO:GSE29264].

## Supplementary Material

Additional file 1 **Tables S1 to S4**. All genome positions and strands are relative to GenBank CP000100. Table S1 - all annotated transcripts: column A, transcript ID number; column B, strand (1 is plus strand, 0 is minus strand); column C, first ORF on transcript; column D, last ORF on transcript; column E, predicted 5' transcription start site; column F, predicted 3' end; column G, length of transcript; column H, is the transcript an mRNA? (all transcripts that included any rRNA or tRNA were not considered as mRNA; Materials and methods); column I, the number of ORFs per transcript; column J, length of 5' UTR; column K, length of 3' UTR; column L, mean of the raw RNA sequencing reads over the full transcript; column M, number of transcripts per cell assuming a total of 1,500 mRNAs per cell. Table S2 - all non-coding transcripts: column A, non-coding transcript ID number; column B, predicted 5' transcription start site; column C, predicted 3' end; column D, strand (1 is plus strand, 0 is minus strand); column E, mean of the raw RNA sequencing reads over the full non-coding transcript; column F, length of non-coding transcript; column G, percent overlap that a non-coding transcript has with an ORF that was designated as not transcribed (designated when mean RNA sequencing coverage of ORF is less than two reads per nucleotide); column H, percentage of non-coding transcript that is antisense to an annotated transcript; column I, does the non-coding transcript pass the high confidence criteria? (Materials and methods); column J, does the non-coding transcript pass the circadian criteria? (Materials and methods); column K, the difference in gene expression of the non-coding transcript in the dusk versus dawn circadian timepoints calculated by tiling microarray (all probes internal to the non-coding transcript were used to make this calculation); column L, RFAM homology. Table S3 - all RNA polymerase peaks: column A, peak ID number; column B, start of peak; column C, end of peak; column D, position of peak maximum; column E, total ChIP reads at peak maximum (sum of circadian timepoints, dawn and dusk, after normalization for total number of reads); column F, *P*-value for enrichment of reads in ChIP sample versus mock immmunoprecipitation. Table S4 - comparison of literature 5' versus RNA sequencing 5': column A, JGI ID for ORF; column B, common name for ORF; column C, strand (1 is plus strand, 0 is minus strand); column D, translation start position of ORF; column E, literature-based 5' transcription start site; column F, alternative 5' transcription start site from literature; column G, 5' transcription start site estimate from our RNA sequencing; column H, difference between our 5' transcription start site estimate and the closest literature estimate; column I, method of 5' transcription start site determination used in the literature reference; column J, literature reference. Table S5 - expression of all JGI predicted ORFs: column A, JGI ID for ORF; column B, *Synpcc7942 *ORF ID; column C, start of ORF (in the case when the ORF is on the plus strand, this is where the start codon is located); column D, end of ORF (in the case when the ORF is on the minus strand, this is where the start codon is located); column E, strand (1 is plus strand, 0 is minus strand); column F, mean of the raw RNA sequencing reads over the full ORF. Click here for file

Additional file 2 **Supplementary Figures S1 to S8**. Figure S1: examples of 5' determination from RNA sequencing. **(a) **5' Determination of the *ntcA *transcript. A sharp drop in RNA sequencing reads is observed at the 5' end of the mRNA. 5' end determination by RNA sequencing and traditional methods [61] differ only by a single nucleotide. **(b) **5' determination of the *purF *transcript. The RNA sequencing estimate is over 80 nucleotides different from that derived by traditional methods [62]. Subsequent experiments [46] have shown that the minimal promoter for the *purF *transcript contains the RNA sequencing 5' end but not the literature 5' end. A more complete comparison of RNA sequencing and traditional transcription start determination is provided in Table S4 in Additional file 1. Figure S2: representative RNA pol ChIP over a 40-kb region. **(a) **RNA sequencing data. Positive strand transcription is shown in blue (positive y-axis), and negative strand transcription in red (negative y-axis). ORFs on the positive and negative strands are indicated by horizontal black lines. RNA pol peaks significantly enriched over the mock immunoprecipitation (*P *< 0.1) are indicated with vertical green lines and those that are not (*P *≥ 0.1) are indicated with vertical pink lines. Large RNA pol peaks tend to be located near the 5' end of transcripts, although there are many peaks in the middle of transcripts potentially caused by RNA pol pausing. **(b) **RNA pol ChIP and mock. RNA pol ChIP (black) and mock immunoprecipitation (green) are normalized such that the genome average is 200 reads per nucleotide. Almost all RNA pol peaks are enriched over the mock immunoprecipitation. A complete listing of RNA pol peaks and their enrichment is provided in Table S3 in Additional file 1. **(c) **RNA pol ChIP normalized by input. Normalization of RNA pol ChIP by input does not qualitatively change the data (compare Figure S2b and Figure S2c in Additional file 2). Figure S3: comparison of changes in gene expression and RNA pol ChIP at two points in the circadian cycle. **(a) **Changes in RNA pol occupancy at two separate times during the circadian cycle (dusk and dawn). Changes in RNA pol are reflective of changes in transcript level by microarray (Pearson correlation, r = 0.6860). The probability of getting a correlation as large by random chance (*P*-value) is 2.2286e-197. Figure S4: characteristics of transcription start. **(a) **Melting temperature at transcription start. The melting temperature of 10-nucleotide fragments from -200 to +200 of all mRNAs was averaged (Materials and methods). A drop in the melting temperature is observed at the promoter. **(b) **Nucleotide content at transcription start sites. Nucleotide content of all mRNAs aligned by transcription start. **(c) **Zoomed in nucleotide content at transcription start. Nucleotide content of all mRNAs aligned by transcription start. Preference for adenine at the +1 position and a -10 element can be observed. Figure S5: comparison of minimum free energy changes with that of dinucleotide-shuffled sequences. **(a) **Minimum free energy change at RNA pol peaks. The minimum free energy of 60-nucleotide RNA fragments with 10-nucleotide spacing was calculated and averaged for all mRNAs (Materials and methods). A drop in minimum free energy slightly prior to the position of the RNA pol peak is observed. To prevent sequence features of the transcription terminus or promoters from interfering with this analysis, a subset of 183 RNA pol peaks satisfying the following criteria were used: (1) RNA pol peak must be closer to a 5' end than a 3' end; and (2) RNA pol peak must be +100 to +300 relative to the 5' end. Since RNA pol ChIP does not specify the strand being transcribed, the strand of transcription was inferred from RNA sequencing data. Dinucleotide shuffled sequences show a qualitatively similar trend to native sequences, suggesting that there is no specific secondary structure at this transition (Materials and methods). **(b) **Sequence changes near RNA pol peaks. A sequence content change from low to high GC content can be observed near the position of the RNA pol peaks. The same subset of RNA pol peaks are used here as in Figure S5a in Additional file 2. A smoothing window of five nucleotides has been applied to smooth nucleotide contents. These sequence changes may be responsible for the free energy changes we observe. It is also possible that these changes in sequence content may contribute to RNA pol pausing by an unknown mechanism. **(c) **Minimum free energy change at transcription terminus. Minimum free energy was calculated as above after aligning all transcripts by transcription terminus. Dinucleotide-shuffled sequences do not resemble native sequences, suggesting that a discrete hairpin-like structure exists at the terminus of transcripts (Materials and methods). **(d) **Minimum free energy change at transcription start. Minimum free energy was calculated as above after aligning all transcripts by 5' transcription start. A drop in minimum free energy occurs globally within transcripts and may be related to our observation of global RNA pol pausing. Dinucleotide-shuffled sequences show a qualitatively similar trend to native sequences (Materials and methods). Figure S6: enrichment in RNA sequencing at 5'. **(a) **Increased RNA sequencing signal at 5' ends. An increase in RNA sequencing signal can be observed at the 5' end of mRNAs. Several biological phenomena may account for this enrichment, but one intriguing possibility is the existence of many partial or nascent transcripts caused by pausing of RNA pol near the 5' end of the transcript. **(b) **RNA pol pausing at 5' ends may contribute to RNA sequencing enrichment at 5' ends. A slight but significant correlation exists between the retention ratio of RNA pol and the enrichment of RNA sequencing prior to the RNA pol peak. The same subset of RNA pol peaks was used as in Figure S5a in Additional file 2. Pearson correlation is r = 0.4591, and the probability of getting a correlation as large by random chance (*P*-value) is 6.2879e-11. Figure S7: the phycocyanin operon - a functional case of partial transcription termination. **(a) ***Partial transcription termination controls the stochiometry of cpcβ *and *cpcα *to rod linker mRNA at approximately 6:1. This stochiometry reflects the organization of the phycobilisome - a hexameric α-β double disc with an associated linker [31]. RNA sequencing data cannot be mapped to the *cpcβ *and *cpcα *coding region because it is not unique in the genome (another copy of *cpcβ *and *cpcα*, corresponding to the core proximal phycobilisomes exists in the genome). The position of predicted terminators (from TransTermHP) is indicated in green, and the position of JGI predicted ORFs is indicated in black. Figure S8: circadian gene expression of putative non-coding RNAs. **(a) **Gene expression by tiling microarray of high-confidence circadian non-coding RNAs. Gene expression of non-coding RNAs with potential for circadian gene expression are plotted by non-coding transcript ID (Table S2 in Additional file 1). Gene expression ratios for non-coding RNAs are computed by averaging the gene expression ratios for all tiling probes internal to the non-coding transcript. Click here for file
