# Supplementary figures and images for: A high resolution map of a cyanobacterial transcriptome
Source: Genome Biol. 2011 May 25;12(5):R47. doi: 10.1186/gb-2011-12-5-r47 (PMC3219970; doi:10.1186/gb-2011-12-5-r47)

A

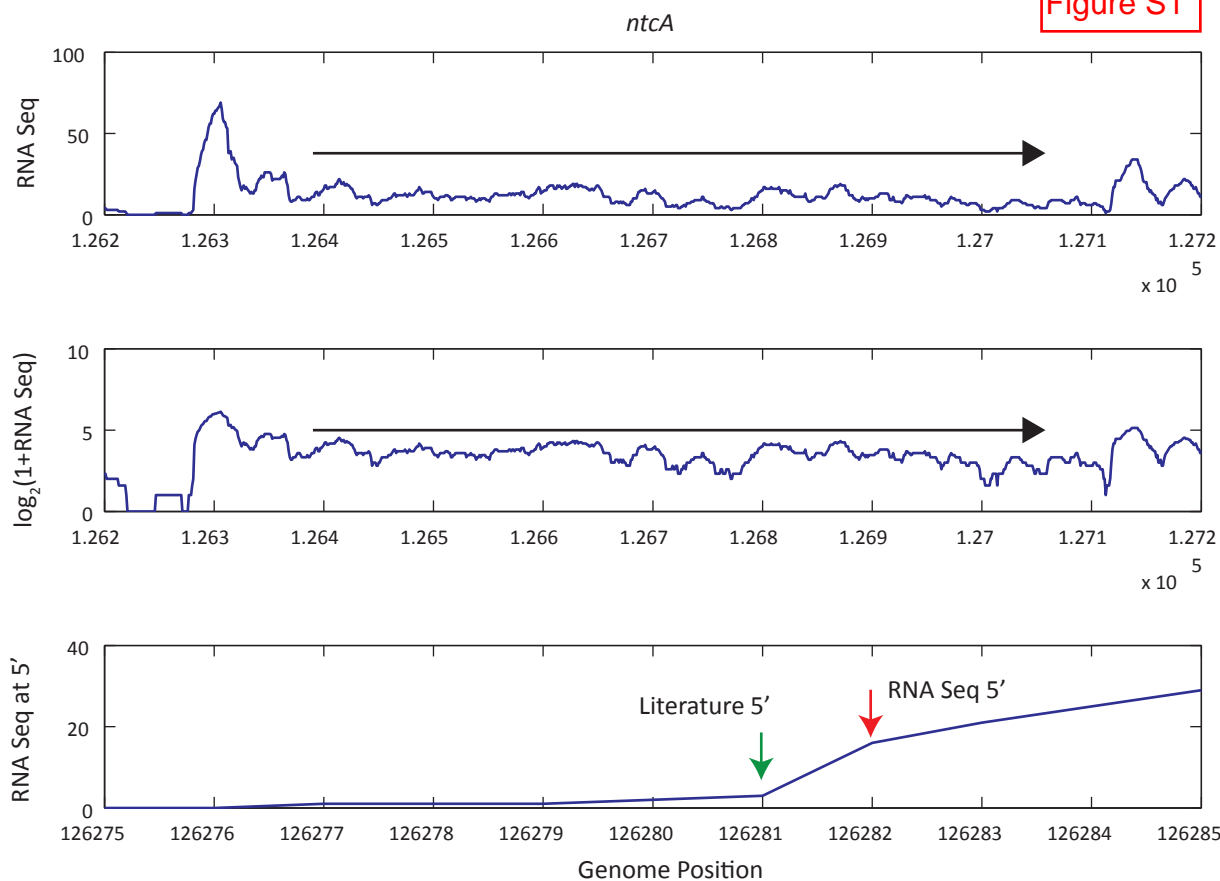

B

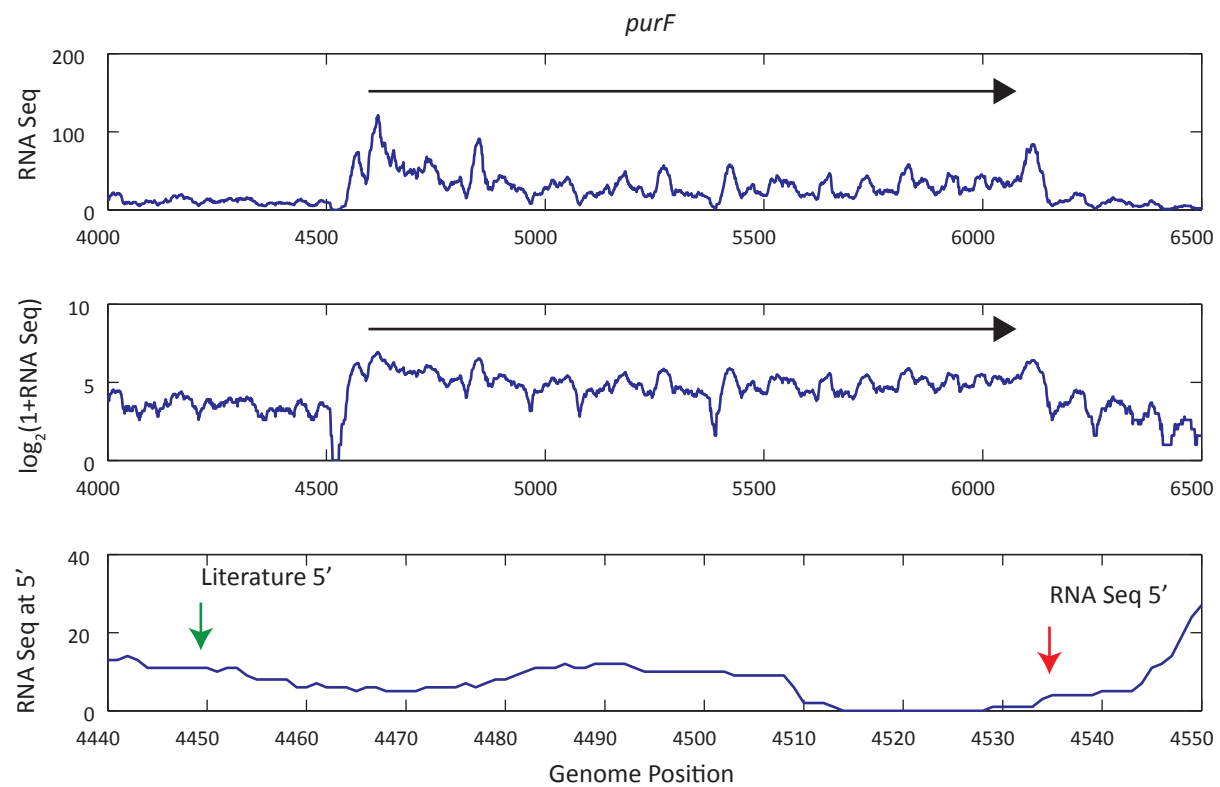

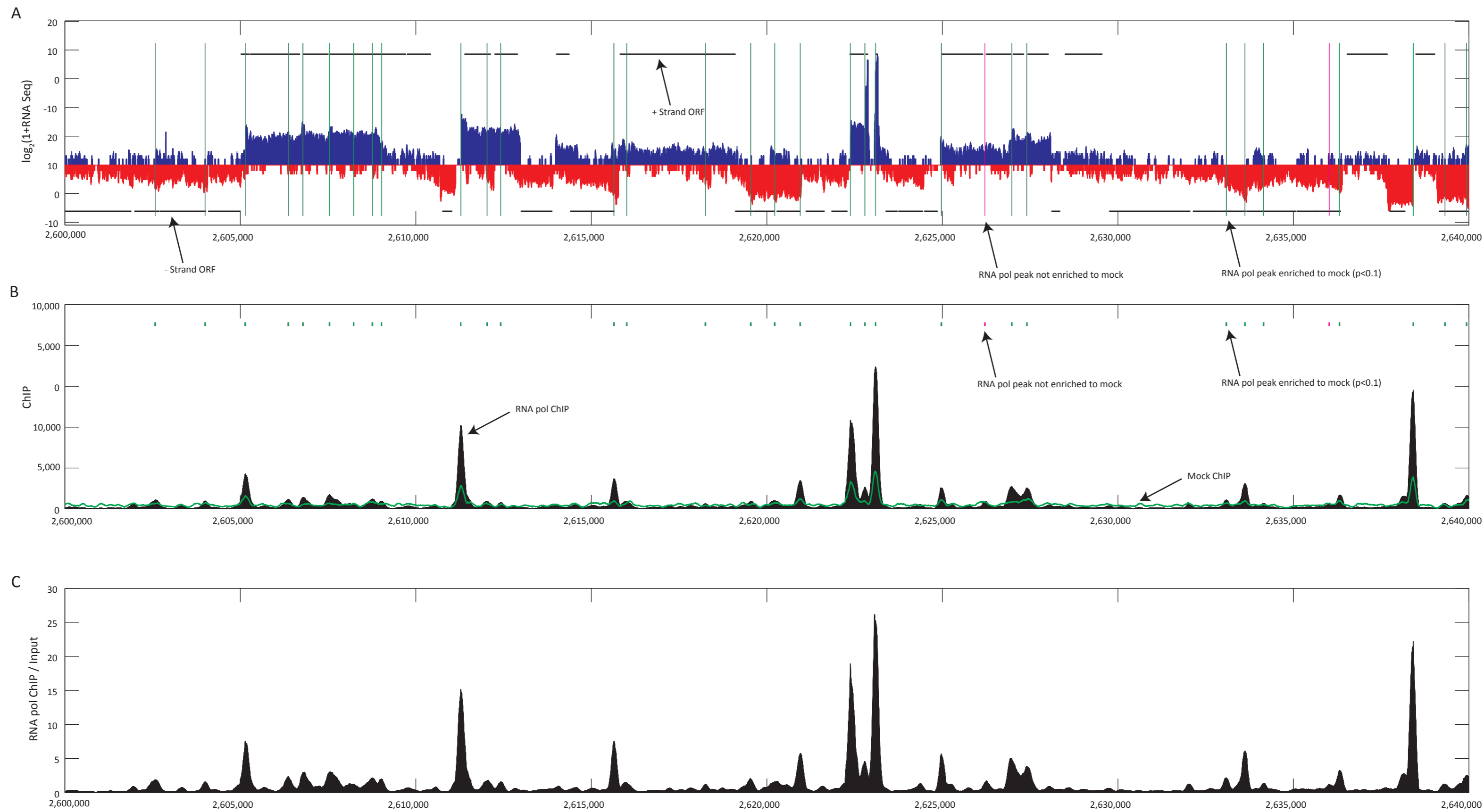

A

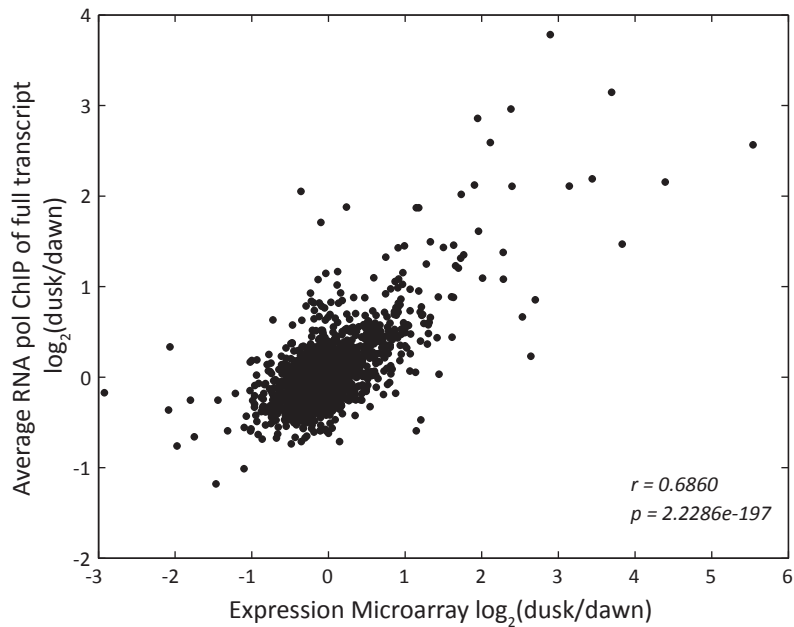

A

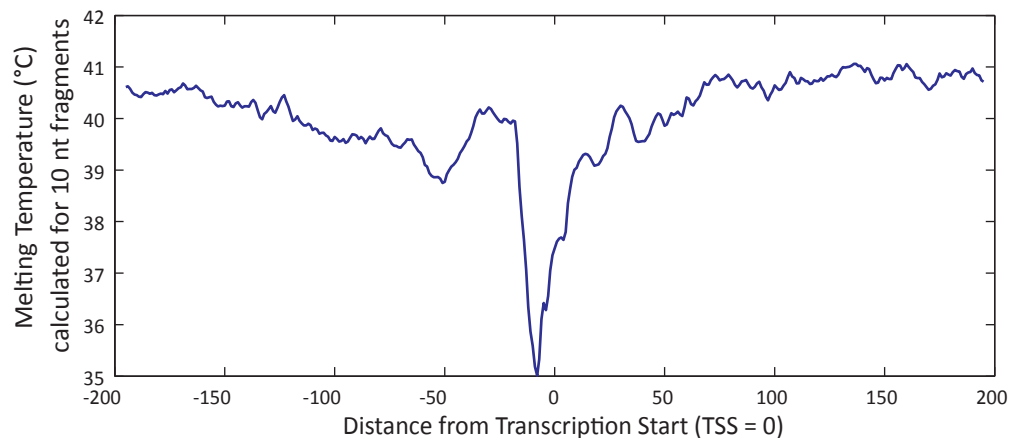

B

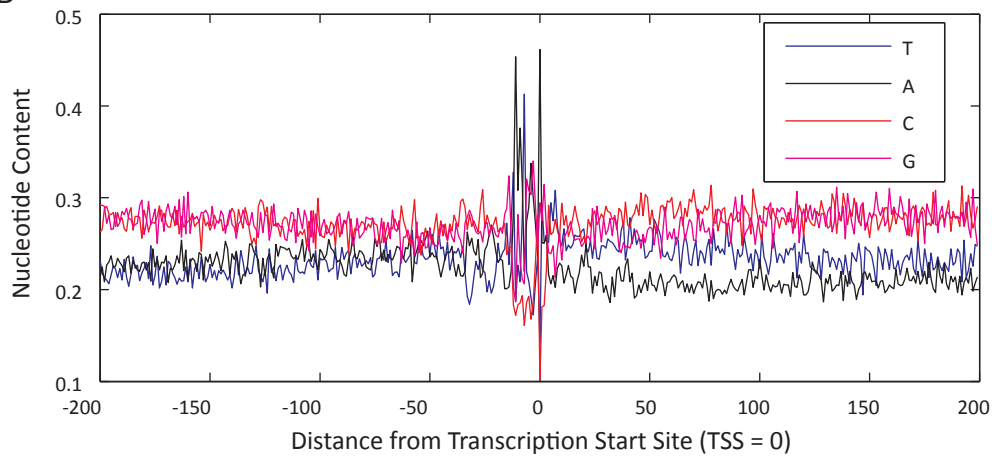

C

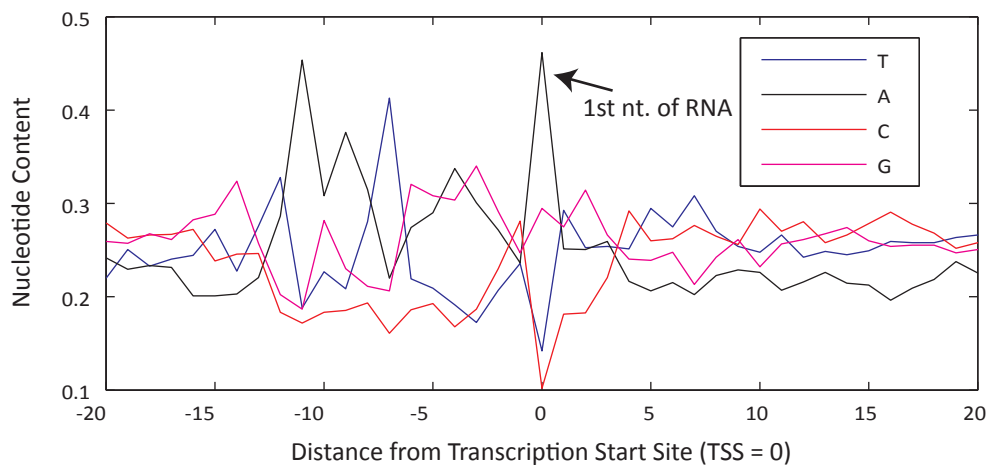

A

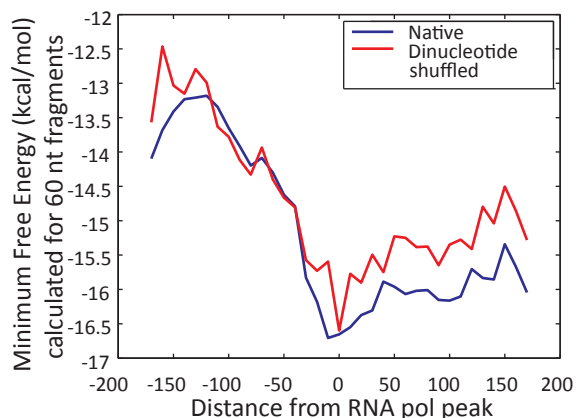

B

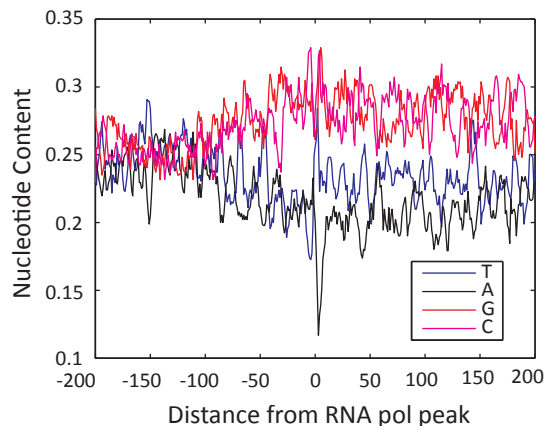

C

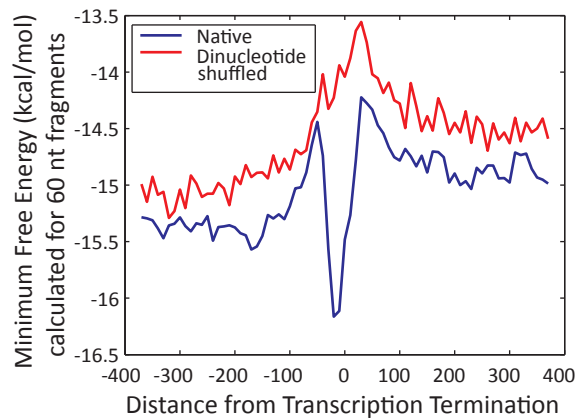

D

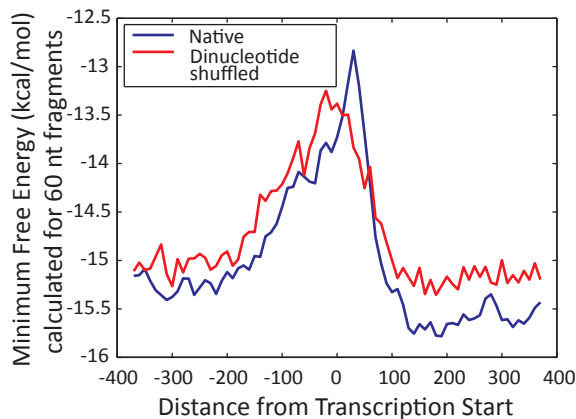

A

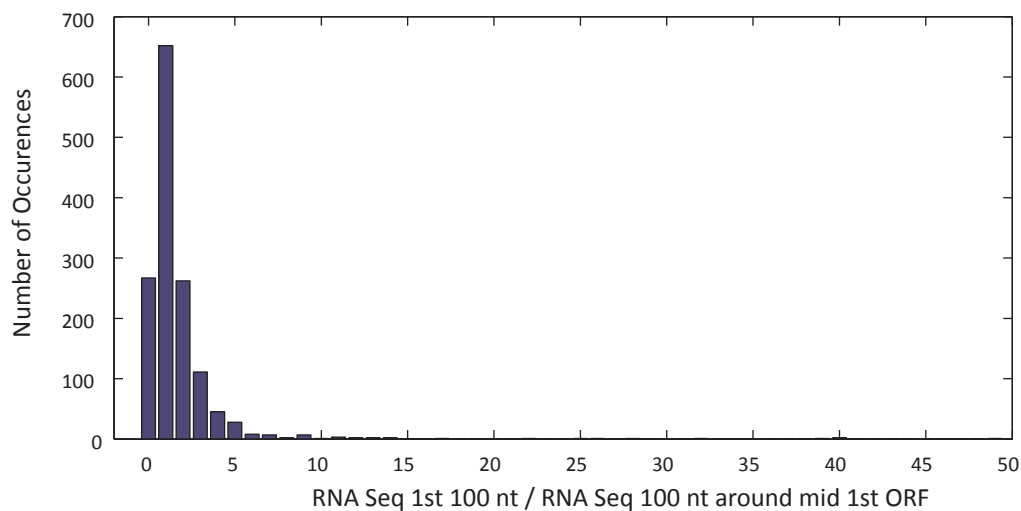

B

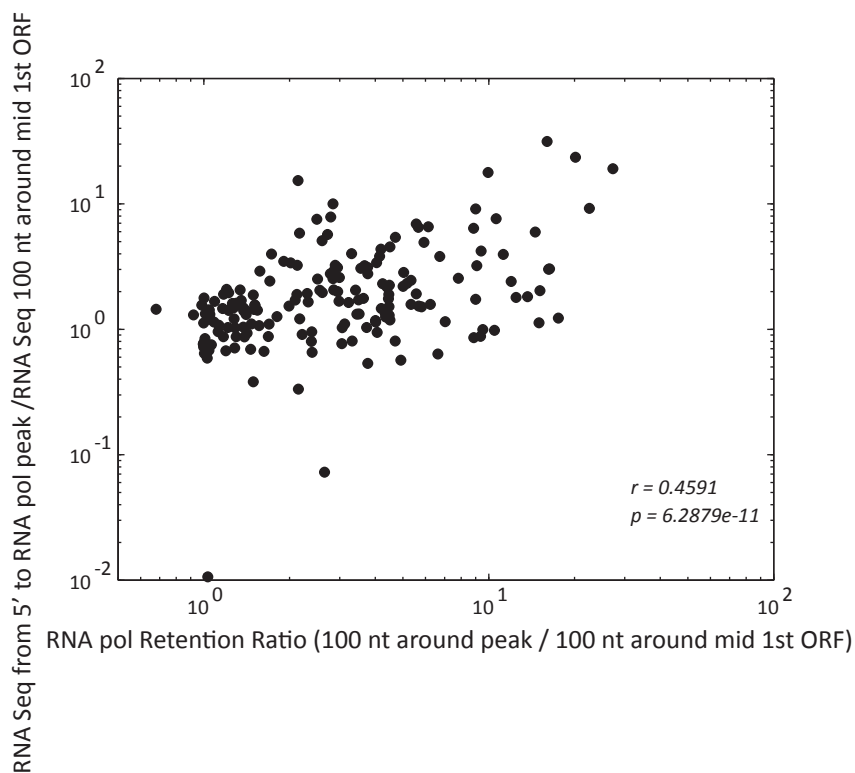

Figure S7

A

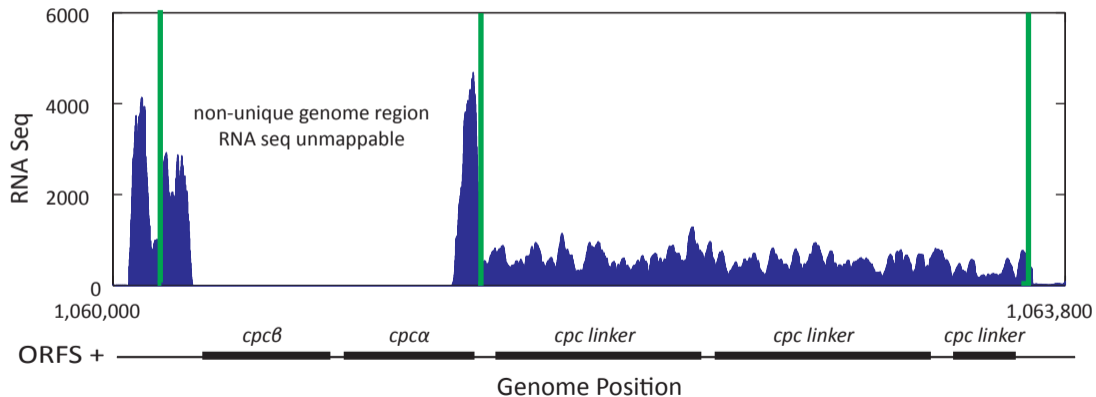

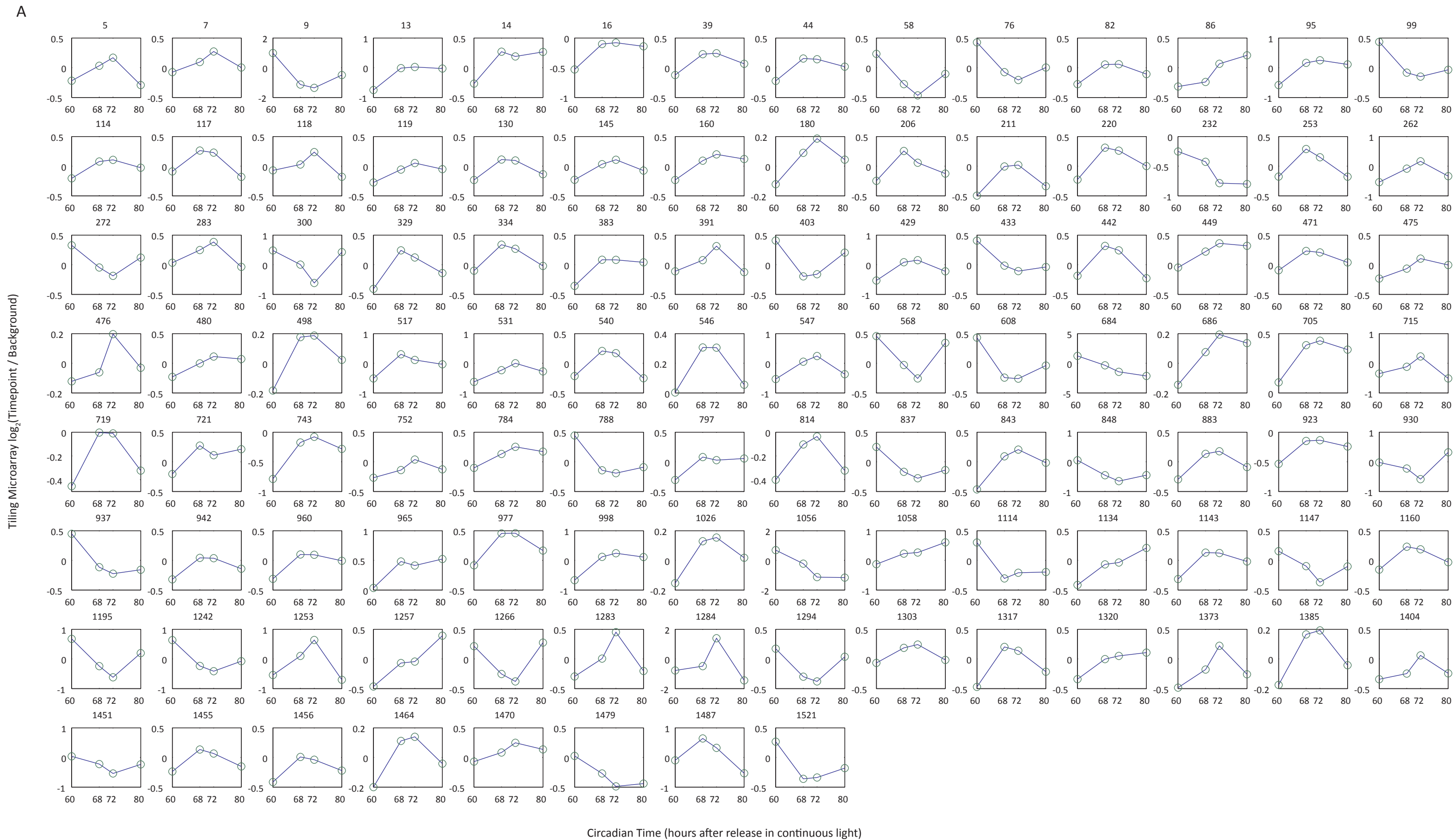

Supplement: Additional file 2 — Supplementary Figures S1 to S8. Figure S1: examples of 5' determination from RNA sequencing. (a) 5' Determination of the ntcA transcript. A sharp drop in RNA sequencing reads is observed at the 5' end of the mRNA. 5' end determination by RNA sequencing and traditional methods [61] differ only by a single nucleotide. (b) 5' determination of the purF transcript. The RNA sequencing estimate is over 80 nucleotides different from that derived by traditional methods [62]. Subsequent experiments [46] have shown that the minimal promoter for the purF transcript contains the RNA sequencing 5' end but not the literature 5' end. A more complete comparison of RNA sequencing and traditional transcription start determination is provided in Table S4 in Additional file 1. Figure S2: representative RNA pol ChIP over a 40-kb region. (a) RNA sequencing data. Positive strand transcription is shown in blue (positive y-axis), and negative strand transcription in red (negative y-axis). ORFs on the positive and negative strands are indicated by horizontal black lines. RNA pol peaks significantly enriched over the mock immunoprecipitation (P < 0.1) are indicated with vertical green lines and those that are not (P ≥ 0.1) are indicated with vertical pink lines. Large RNA pol peaks tend to be located near the 5' end of transcripts, although there are many peaks in the middle of transcripts potentially caused by RNA pol pausing. (b) RNA pol ChIP and mock. RNA pol ChIP (black) and mock immunoprecipitation (green) are normalized such that the genome average is 200 reads per nucleotide. Almost all RNA pol peaks are enriched over the mock immunoprecipitation. A complete listing of RNA pol peaks and their enrichment is provided in Table S3 in Additional file 1. (c) RNA pol ChIP normalized by input. Normalization of RNA pol ChIP by input does not qualitatively change the data (compare Figure S2b and Figure S2c in Additional file 2). Figure S3: comparison of changes in gene expression and RNA pol ChIP [file gb-2011-12-5-r47-S2.PDF]
